# Practical Remarks on Blood-Letting

**Published:** 1866-07

**Authors:** D. B. Trimble

**Affiliations:** Chicago, Ill.


### CHICAGO MEDICAL EXAMINER.

N. S. DAVIS, M.D., Edtor.

VOL. VII. JULY, 1866. NO. 7.

### Original Contributions.

### ARTICLE XXII.

### PRACTICAL REMARKS ON BLOOD-LETTING.

[CONTINUED.]

By D. B. TRIMBLE, M.D., Chicago, Ill.

The next portion of the subject that will claim attention will be that of diseases affecting the digestive system.

In wounds or other injuries of the parieties of the abdomen, or of the abdominal viscera, venesection is often required to subdue or moderate inflammation.

In many of the diseases of the alimentary canal, (both extra and inter-abdominal,) especially those of an inflammatory nature, blood-letting, general and local, is frequently necessary.

*Stomatitis.*—In the various inflammations of the mucous membranes of the mouth, there is but one variety—the common diffused inflammation—in which blood-letting is required; and then only in severe cases, and locally, by leeches.

*Glossitis*, or inflammation of the muscular portion of the tongue, is a disease so dangerous in its tendencies, and so rapid in its termination, as often to require the promptest and most active treatment. The inflammation is of the sthenic type, as shown by the strong and frequent pulse, and hot skin; and asphyxia, apoplexy, or a gangrenous condition of the tongue may end the case fatally, if not relieved. No remedy acts so promptly and efficiently in such cases as free venesection (followed by leeches to the lower jaw or to the neck below it) repeated until the inflammation is lessened or controlled. When these measures, and other remedial means that may be deemed necessary, do not succeed, free incisions into the tongue may give relief.

In *Angina*, or ordinary sore throat, bleeding is very seldom necessary, but if the disease is severe and accompanied by fever, headache, and a full, strong pulse, moderate venesection may be required. In the *diphtheritic* variety of angina, some authors recommend venesection and leeching, but I have never resorted to it in this disease, and do not favor its use. This affection, if seen in the early stages, is generally easily controlled by other and milder means; and if not seen until the disease is fully developed, the prostration is usually so great as to preclude its use.

*Tonsillitis or Quinsy*, if seen during the forming stage, may frequently be prevented from proceeding to suppuration by active venesection, followed, if necessary, by leeches to the upper anterior portion of the throat. By this treatment I have sometimes succeeded in preventing the tonsils from suppurating in persons subject to frequent attacks of quinsy. If the patient is aged or feeble, or the fever light, it should be omitted. If venesection or leeching are not required, scarifying the tonsils will afford considerable relief.

In *Pharyngitis and Œsophagitis*, if of the sthenic grade, with a strong, full pulse, bleeding, both local and general, is indicated, though many cases will do well without it. These phlegmasiæ generally terminate in resolution, but sometimes abscesses form, and are the cause of much danger. On this account, chiefly, are antiphlogistic remedies necessary.

*Gastritis* seldom occurs as an idiopathic affection, but is generally an attendant on some other disease, or is produced by the introduction into the stomach of poisons, or acrid or corrosive substances. When idiopathic and acute, it requires depletion as freely as the phlegmasiæ of the viscera generally; and, also, when an attendant on other diseases, venesectiom may sometimes be indicated, though generally not to so great an extent. When caused by poisons or corroding substances, the system is usually too prostrate to bear general depletion; though after reaction (when it occurs), leeches applied to the epigastrium have sometimes a beneficial effect. In those cases, also, which are consequent on other diseases, leeching the epigastrium will often allay the vomiting and pain, and lessen the inflammation. They should also be used in idiopathic cases, after sufficient venesection has been practised. HABERSHON and BRINTON advocate leeching only. In the *chronic* form, venesection is seldom required, unless there is fever, with a full and strong pulse, when a moderate bleeding will often alleviate the symptoms. A few leeches applied over the epigastrium and often repeated, will frequently accelerate the cure.

In *spasm* of the stomach, if there is inflammatory action, venesection may be necessary, or cups or leeches applied to the epigastrium; or, if depending upon spinal ittitation, to the spine.

In *Gastric Irritation*, arising from the same cause, the same treatment is indicated.

In *Ulceration* of the Stomach, HABERSHON recommends leeches applied to the scrobiculus cordis; while BRINTON admits that they will afford temporary relief, but does not advocate their use. To be of any permanent benefit, they should, no doubt, be applied early, and while the ulceration is superficial. In *chronic* ulceration, and in the other organic and functional diseases of the stomach, as tumors, cancers, dyspepsia, etc., it is rarely, if ever, that depletion is indicated.

In *Cholera Morbus*, though some authorities recommend general or local bleeding, it is not, in my opinion, ever required, unless symptoms of gastritis should supervene, when leeches to the epigastrium may be beneficial.

Blood-letting is one of the numerous remedies recommended in the treatment of *Asiatic Cholera*, on which subject there is much diversity of opinion. The remarks that I may make relating to this disease I will quote chiefly from an article contributed to the April number of the *Chicago Medical Journal*, for 1866. "Dr. George Johnson, Prof. of Medicine in Kings College, takes the position that the collapse of cholera is not due to a drain of liquid from the blood, and strongly advocates free venesection as the remedy chiefly to be relied upon" in many cases. In regard to the effect of venesection on the symptoms of collapse, he says:—"But what has been the effect of venesection in not one or two, but in a large number of cases of cholera, and in the hands of many practitioners?" It is in the writings of the Indian practitioners, that the largest amount of evidence is to be obtained, as to the influence of blood-letting in cholera. Quoting from them, he says:—"Annesley says, in place of syncope being produced by bleeding in the cases which I have treated, the pulse has invariably improved, and the feelings of faintness and debility disappeared." "Bell makes the following statement:—The effect of blood-letting would, indeed, sometimes appear almost miraculous. A patient will be brought in on a cot, unable to move a limb, and, but that he can speak and breathe, having the character, both to touch and sight, of a corpse; yet will he, by free venesection alone, be rendered, in the course of half-an-hour, able to walk home with his friends." After giving still further testimony to this treatment, he says:—"I maintain that the numerous well-authenticated instances of great, and immediate, and permanent relief by means of venesection in the collapse of cholera, are irreconcilable with the hypothesis in question, *viz.*, the loss of liquid constituents of the blood." But Dr. Wood advises it only "in those rare cases in which the pulse is full and strong, especially if connected with convulsive symptoms, blood should be taken from the arm." In my father's case, referred to in the same article, the system was in the condition referred to by Prof. Johnson, the "surface was cool and shrivelled, and the pulse at the wrist almost imperceptible," and this was his physician's reason for bleeding, I suppose on the theory of internal congestion. "Leeching is recommended in some cases, both by Prof. Wood and Dr. Aitken. The former advises it in the earlier stages to the epigastrium, where there is tenderness or burning pain in the stomach; the latter, to the temples, when there is headache." In my own practice I have seldom resorted to depletion, but, from the effects that I have seen produced by it, and the testimony of medical writers to its efficacy, I hope to see it tested, if the disease should appear in our midst.

In *Cholera Infantum*, if there is much tenderness of the abdomen, and not too much depression of the system, leeches may be applied to the epigastrium, sometimes with a happy effect.

The diseases of the duodenum are so similar to those of the stomach, that the treatment applicable to the former is, to a great extent, applicable to the latter. *Duodenitis* and *congestion* of the mucous membrane of the duodenum are the only diseases of this organ in which direct depletion is required.

In *acute Enteritis*, where either the mucous membrane of the intestine only, or all of its coats are inflamed, if the constitution is good, and the pulse full and frequent, venesection is indicated, and generally relieves the constipation which is a frequent effect of this disease, and moderates the pain and the violence of the inflammation. If the disease should occur in the feeble or aged, or in a mild form, bleeding is not required. Leeching or cupping the abdomen over the region of the small intestines should be resorted to when the inflammation is severe and venesection not admissable, or should succeed venesection. In the *chronic* form, general bleeding is seldom required, but leeches are sometimes very beneficial.

The *Cœcum* is also the subject of inflammatory action, a frequent cause of which is its peculiar form, in which, sometimes, the intestinal contents are impacted, giving rise to irritation and inflammation of its coats, and occasionally terminating in ulceration or suppuration. Some years ago, I was called in consultation in a case where the attending and consulting physicians could not agree in regard to the exact nature of the disease. I found the patient with an abscess in the right iliac region, which had opened externally, and from which passed, beside pus, watermelon seeds, fish-bones, etc. He was emaciated, skin of an icterode hue, etc. The attending physician, a young man, was of the opinion that it was a case of hepatitis, resulting in the formation of an abscess, and that by adhesion between the liver and duodenum a portion of the contents of the latter had passed through the liver, and from the body by its opening through the abdominal wall. The consulting physician, an old and experienced practitioner, was of the opinion that the case was one of typhlitis, caused by the seeds, etc., that had lodged in the cæcum or appendix vermiformis, and that this was the point from which they were ejected. With thir view of the case, I concurred, and the patient dying the next day, gave the opportunity, by a necroscopic examination, to determine the question. The consulting physician's view was fully confirmed, by the appendix vermiformis being found perforated, but still containing some of the seeds, etc,, similar to those thrown off, intermingled with pus. In typhlitis from this cause, depletion could have only a palliative effect; but when produced by cold or any of the ordinary causes, depletion is as beneficial as in enteritis. Professor JACKSON, of Cambridge, relates some cases which he denominates "painful tumors *near* the cæcum, and in which the cæcum may be suspected to take a part." It is characterized by a tumor and severe pain in the right iliac region; in some cases there is fever and constipation, and the abdomen is painful, hard, and tender, when free venesection and leeching promptly alleviated the sufferings, and the cures were generally rapid. In mild cases, leeching only was resorted to.

In an article on the treatment of *Dysentery*, written for the *Chicago Medical Journal*, August, 1865, I stated that during two extensive and severe epidemics that prevailed, "venesection was seldom resorted to; and the few instances in which I did bleed were in persons of good constitutions, in the vigor of life, and where the fever ran high, and in these cases with much benefit." "Though there are diseases in which I would resort to venesection, where many of the physicians of the present day would not, yet dysentery is not one of those diseases, except in peculiar circumstances. The disease often produces great debility, and is sometimes prolonged to several weeks, so that it is necessary to economize the strength of the system as much as possible; and unless the inflammation of the colon and rectum is of a very active grade, we can generally succeed without it." Dr. WOOD says:—"Bleeding should always be resorted to when there is much pain and tenderness of the abdomen, with febrile action and a vigorous pulse," and, "if the local symptoms are unabated, and the pulse remains firm, may be repeated again and again." On the other hand, HABERSHON remarks:—"I have never observed a case of dysentery, where venesection could have been tried with probable success." I have had much experience in the treatment of this affection, and think one author about as far wrong as the other. I do not remember to have bled in any case more than once, and have attended many cases where there was "much pain and tenderness of the abdomen, with febrile action" that have done well without this measure; and on the other hand, I have resorted to it in cases in which the cure, I am confident, was much accelerated by it, and in which success might not have been attained had it been neglected. I concur, however, with the latter author in his further remarks, that "local depletion is of great service, either applied to the anterior surface of the abdomen or to the anus. The latter more directly acts on the intestine, on account of the connection of the inferior hemorrhoidal with the inferior mesenteric veins." In *chronic* dysentery, if there is much pain and tenderness, leeches may also be applied to the abdomen. In those acute cases, where there are typhoid symptoms or those of an adynamic character existing, venesection cannot safely be resorted to, and but seldom local bleeding.

*Diarrhœa* is a disease of such varied character, and depending on so many different causes, as to make it difficult to follow any regular line of practice. The only cases in which bleeding is called for, are those of an inflammatory or bilious character, when moderate bleeding, general or local, may be necessary.

In *Colic*, bleeding is recommended by some authorities as useful in relaxing the spasm in that variety of the disease, and also in reducing inflammation. I do not remember ever to have bled in this affection. In bilious, rheumatic, and inflammatory colic, venesection may be employed still more freely than in spasmodic colic, especially in the latter two varieties. In bilious colic, the addition of cups to the right hypochondrium will be beneficial.

In *Constipation*, arising either from inflammation, spasmodic action, or obstruction of the bowels from any cause, venesection should be freely practised with the view of alleviating the inflammatory condition, or producing relaxation. Cupping or leeching the abdomen, or leeches applied to the anus, may be used in addition.

In *Hemorrhoids*, where the system is plethoric, venesection is sometimes beneficial, but is seldom necessary. Leeches applied to the anus, or scarifications of the tumors are often highly useful. Since my residence in this city, I have had one severe case of piles, of long continuance, cured, at the time, chiefly by the application of half-a-dozen leeches and the recumbent posture. I had previously scarified the parts with but slight benefit. The attacks since, have been very light and infrequent.

In *Prolapsus Ani*, if the bowel cannot be reduced on account of inflammation and tumefaction, or constriction of the sphincter, venesection will be indicated, followed, if the inflammation and spasm are not subdued, by leeches to the nates.

For similar reasons, venesection is often required in *Hernia*, particularly if strangulated. It will sometimes so far relax the stricture, and reduce the size of the protruded intestine, as to produce a voluntary reduction; and if not, renders the taxis more speedily and safely successful, and lessens the danger of subsequent inflammation.

In *Peritonitis* of the acute and puerperal forms, prompt and active depletion is the sheet-anchor of hope. Full venesection at the commencement of the disease, repeated once or more, if necessary, and followed by the application of leeches to the abdomen, can seldom be omitted without great risk to the patient. Nor can the pulse or countenance be taken for the guide for this remedy in this disease, for the pulse is generally small and very frequent, though corded, and the face pale. The pulse is often rendered freer, fuller, and slower by venesection, and when this is the effect, it may be continued without fear of evil consequences. This treatment applies to mesenteritis, omentitis, or other partial inflammations of the peritoneum, equally, though modified in degree, as to peritonitis. In *chronic* peritonitis, leeching the abdomen occasionally, is sometimes required.

In acute inflammation of the *Mesenteric Glands* either as the result of enteritis, or connected with a strumous condition of the glands, leeches or cups may be applied to the abdomen with the view of preventing the formation of abscesses, or to retard the progress of the disease when tuberculous.

In many diseases of the accessory organs of digestion, direct depletion is often required-

In some diseases of the *Liver*, it is frequently of very great benefit.

*Hepatitis.*—The phlegmasiæ of the liver, arising from various causes, affecting different portions of the viscus, differing in their characters, and producing different effects, cannot properly be treated of under one general head. Dr. BUDD, in his work on "Diseases of the Liver," classifies them as "suppurative," "gangrenous," and "adhesive inflammations," "inflammation of the veins of the liver," and "of the gall, bladder, and ducts." While Dr. WOOD, in his work on "Practice," in more general terms, considers it as affecting "the substance of the liver, its investing peritoneal membrane, or both;" or involving "the whole organ, or only a part." As my object is to treat of one only of the remedies applicable to this disease, (as well as to others,) I cannot enter into an elaborate consideration of the various forms of inflammation, in this connection. In suppurative inflammation, if caused by external violence to the liver, or by ulceration of the stomach, intestines, or gall-bladder, BUDD recommends depletion, especially local, whereby the extent of the inflammation and abscess may be lessened, and, in some cases, the latter be prevented from forming. But if caused by phlebitis, consequent upon injuries to the head or limbs, "the whole mass of venous blood is contaminated by pus," a typhoid condition is soon induced, and bleeding is contra-indicated. BUDD lays much stress on the danger of opening abscesses of the liver, and he is probably correct, as his experience in this class of diseases was very great; but in the few cases that I have attended, I have had no unfavorable symptom following the operation as its sequent. Twelve or fifteen years ago, I attended a middle-aged man, who was reduced to a state of extreme prostration and emaciation by a severe attack of hepatitis, terminating in the formation of a very large abscess, which *pointed* in the lower portion of the right hypochondrium. I lanced it, and an immense amount of pus was discharged, from which time he began to improve, and was slowly, but entirely, restored to health. One of the principal effects of *adhesive* inflammation of the liver, is the production of *cirrhosis*, which soon terminates, if not relieved, in an atrophied condition of the organ. If the disease is seen at its commencement, which, from its insidious character is not often the case, among the most efficient remedies are cupping or leeching over the region of the liver. "While there is much tenderness, and some degree of fever exists, nothing produces so much relief as the local abstraction of blood." (BUDD.) This remedy must be more cautiously used in the cases of the habitually intemperate, and, unfortunately, the use of alcoholic drinks is the most frequent cause of the disease. There are other forms of hepatitis beside those already considered, arising from other causes, as when the result of pneumonia, scarlatina, etc., and where antiphlogistic measures are indicated. In acute hepatitis, when the pulse is full and strong, and the constitution good, without having regard to the peculiar character of the inflammation, free venesection should generally be practised, and repeated if the symptoms are not alleviated. "Local bleeding is an excellent auxiliary to the lancet, or substitute for it when forbidden. It is very rarely indeed that, in acute hepatitis, blood cannot be taken with propriety from the vicinity of the disease." (Dr. WOOD.) In *chronic* hepatitis, local depletion alone is required, and that only occasionally. In inflammation of the *biliary ducts*, and of the *gall-bladder* and *ducts*, leeching the right hypochondrium or epigastrium, and, occasionally, general bleeding is indicated. "Leeches relieve the pain and tenderness, and mitigate the inflammation, and, in consequence, lessen the danger of perforation and of permanent closure of the ducts." (BUDD.)

In *Congestion* of the liver, local, and sometimes general, bleeding may be necessary, but in the great majority of cases these measures may be omitted, other remedies being sufficient.

In *Biliary Calculi*, venesection is sometimes beneficial, by relaxing the spasm of the duct and enabling the calculi to pass; and also in subduing inflammation. If the patient is robust, and not too much prostrated, this measure may greatly aid the cure, but often the prostration is so great as to contra-indicate it. If there is much tenderness after the passage of the calculi into the duodenum, leeches should be applied over the region. Though not strictly a portion of our subject, it will, perhaps, not be deemed intrusive if I relate a case in which death was caused by a gall-stone. The patient was a middle-aged married woman, who had been in ill health for several years, with symptoms of indigestion, and was eventually confined to her room for months. She had frequent attacks of vomiting, her food returning often undigested, and was generally constipated. Sometimes she had diarrhœa, but no bile, either in the substances vomited, or in the dejections. Her skin was slightly tinged with an icterode hue, more like its appearance in cancerous affections than in jaundice; there was a small tumor in the epigastric region, and some tenderness. She became greatly emaciated, and I thought her disease was scirrhus of the pylorus. But the necroscopy exhibited an undiseased, though greatly contracted, stomach; and, in examining the liver, I discovered a calculus as large as a pigeon's egg, embedded in its substance where the choledoch duct *should have been*, but of which there was not a trace. The connection between the gall-bladder and duodenum was completely obliterated; of course, no bile could pass into the latter, and digestion and life were eventually destroyed. Another singular *absence* in this case, (though having no bearing on our subject,) was that of the uterus. There was no trace of it, and no appearance indicating that it had ever existed.

*Jaundice.*—In those cases of jaundice where there is tenderness of the right hypochondrium, or of the duodenum, leeches applied to either part, respectively, may afford much relief. In some cases, especially if there is much arterial excitement, it may be necessary to take blood by venesection, but the great majority of cases do not require it. Drs. BUDD, WOOD, and JACKSON, of Cambridge, especially the latter, recommend leeching in this disease. Though admitting its propriety where there is vascular irritation of the liver, gall-ducts, or duodenum, I have seldom resorted to local, never, I believe, to general depletion in jaundice, believing it to be rarely indicated by the symptoms.

Of the diseases of the Pancreas, *Acute Pancreatitis* is the only affection that requires general bleeding, though in *Chronic Pancreatitis*, as in the acute, and in *Cancer* of this viscus, when inflammation exists, leeching is beneficial. Just before my removal to Chicago, I lost an old patient and friend in Philadelphia, of cancer of the pancreas, the only case I ever saw. His disease commenced, (or rather the first symptoms indicating it,) with an inability to retain his food, which, in the course of a few weeks, merged into active and severe emesis, severe lancinating pain in the epigastrium, a deeply jaundiced appearance of the skin, and great emaciation, terminating in death in about four monthsafter the symptoms were developed. The diagnosis in his case was not clear, and his physicians were in some doubt of the exact nature of his disease until the autopsy revealed it.

Though the functions of the spleen are not certainly determined, yet its situation in the abdomen, and proximity to the most important organs of digestion, appear to render this the proper place to consider the diseases to which this organ is subject, and which require depletion.

In *Acute Splenitis*, both general and local bleeding are required in the majority of cases. Where there is great debility, or in the chronic form, this treatment should be restricted to leeches applied to the left hypochondrium. I have frequently attended cases of splenitis, and where I have resorted to depletion, found it to relieve, promptly, the pain, lessen the tumefaction, and facilitate the cure. I was once called, in the absence of his physician, to a young man whom I found in a dying condition, and who died in a few hours. The next day, (his physician not having returned,) I was summoned by the coroner, in connection with another physician, to hold a *post mortem* examination, as it was supposed, from circumstances, that he had been beaten to death. With the exception of a slight bruise on one temple, (which might have been caused by a fall,) there was no external mark of injury. On opening the abdomen, we found a large quantity of blood in its cavity; and on examining the spleen, there were two ruptures disclosed, each crucial in form, and the spleen (which had apparently been much enlarged) was shrivelled or contracted. The patient had formerly, and but a few months before, had intermittent fever, and lived in a miasmatic region. Whether the engorged spleen had been ruptured by a fall or a blow, it was difficult to decide; but the absence of any external mark over its region inclined us to the former opinion. I suppose it was a case of chronic inflammation, enlargement, and softening, induced by malarial influences.

In some cases of *Congestion* of the spleen, if the constitution is robust and the disease active, bleeding may be resorted to; but it is not generally required.

*June 6th, 1866.*

